# Comprehensive Assessment of Artificial Intelligence Tools for Driver Monitoring and Analyzing Safety Critical Events in Vehicles

**DOI:** 10.3390/s24082478

**Published:** 2024-04-12

**Authors:** Guangwei Yang, Christie Ridgeway, Andrew Miller, Abhijit Sarkar

**Affiliations:** Virginia Tech Transportation Institute, Blacksburg, VA 24061, USA

**Keywords:** driver monitoring, driver gaze analysis, driver state monitoring, safety critical events, crash risk analysis, collision warning, computer vision, machine learning, deep learning, artificial intelligence

## Abstract

Human factors are a primary cause of vehicle accidents. Driver monitoring systems, utilizing a range of sensors and techniques, offer an effective method to monitor and alert drivers to minimize driver error and reduce risky driving behaviors, thus helping to avoid Safety Critical Events (SCEs) and enhance overall driving safety. Artificial Intelligence (AI) tools, in particular, have been widely investigated to improve the efficiency and accuracy of driver monitoring or analysis of SCEs. To better understand the state-of-the-art practices and potential directions for AI tools in this domain, this work is an inaugural attempt to consolidate AI-related tools from academic and industry perspectives. We include an extensive review of AI models and sensors used in driver gaze analysis, driver state monitoring, and analyzing SCEs. Furthermore, researchers identified essential AI tools, both in academia and industry, utilized for camera-based driver monitoring and SCE analysis, in the market. Recommendations for future research directions are presented based on the identified tools and the discrepancies between academia and industry in previous studies. This effort provides a valuable resource for researchers and practitioners seeking a deeper understanding of leveraging AI tools to minimize driver errors, avoid SCEs, and increase driving safety.

## 1. Introduction

Road users have been identified as a sole or contributing factor in 94% of crashes in the US and in 95% of crashes in the UK [1]. Further, human factors such as speeding, inattention, distraction, and performance errors were found to be a contributing factor in 92.6% of all crashes [2]. These findings indicate that drivers’ behavior is the most important factor in traffic safety compared to other vehicle or roadway engineering factors. The National Highway Traffic Safety Administration (NHTSA) works to eliminate risky driver behaviors, such as drunk driving, drug-impaired driving, distracted driving, infrequent seat belt usage, speeding, and drowsy driving, on the nation’s roads [3].

To identify and mitigate risky driver behavior, a Driver Monitoring System (DMS) is a critical component of behavioral-change models. A DMS can help minimize driver errors and alert drivers when they have reduced levels of perception or decision-making capabilities in order to reduce the probability of traffic accidents for human-driven vehicles (Level 0) to fully automated vehicles (Level 5) [4]. OEM-integrated DMSs are especially necessary for Level 2 and Level 3 vehicles that require a driver to take over control of the vehicle in certain scenarios. These systems are typically provided by Tier 1 and Tier 2 suppliers then integrated directly into the vehicle systems. Separately, many aftermarket DMSs are used in occupational settings to track and analyze driver behavior and vehicle performance in real time to ensure safe and efficient operations and minimize crashes [5]. These aftermarket DMS typically provide their own platform by which managers can track driver behaviors. Accordingly, both types of DMS can help auto makers and industry technology vendors develop safety methods considering the current driver state and readiness.

Furthermore, DMSs integrated into the vehicles using OEM primarily serve to alert drivers through the vehicle-based approach; however, recent developments towards Level 2 and Level 3 deployments have begun integrating driver-facing cameras to detect fatigue or distraction. Similarly, aftermarket DMSs have routinely utilized these cameras, though they have only recently begun to include algorithmic evaluation of driver behaviors. Traditional implementation of aftermarket DMSs has been at an organizational level to capture and report behaviors of employed drivers to management [6]. These systems aggregate behaviors within and across drivers to track drivers’ locations and routes, produce driver scorecards, identify risky drivers, or evaluate organizational factors such as idle time or fuel costs. These scorecards are typically provided to management through a portal to supply detailed driving patterns for coaching driver behaviors. At the organizational level, DMSs are most often influential at reducing risky driving behaviors only when feedback is included through some form of supervisory coaching or managerial accountability [7].

A DMS typically monitors drivers’ behavior via three approaches: vehicle-based, physiological, and behavioral. The vehicle-based approach tracks data from vehicle components, such as steering wheel, seat belt, brake pedal, road-facing cameras, Global Navigation Satellite System (GNSS), Inertial Measurement Unit (IMU), etc., to detect abnormal driving patterns or to estimate driver behaviors [8,9,10]. This is a non-intrusive approach to drivers, but is challenging for real-time monitoring of risky driver behaviors. The physiological approach attaches sensors to drivers to obtain human body signals (heart rate, skin conductance, respiration rate, skin temperature, etc.) for DMSs [8,11,12]. This approach is used to detect certain driver states, such as fatigue or stress; however, drivers often consider the attached sensors to be intrusive. Lastly, the behavioral approach captures driver-facing video recordings to manually or algorithmically assess specific driver behaviors [13,14,15].

The last decade has seen enormous advancement in Artificial Intelligence (AI) to optimize computing power, big data analysis, Machine Learning (ML), and Computer Vision (CV). Also, enhanced sensor affordability and efficiency have increased the reliability and cost-effectiveness of automation. In particular, Deep Learning (DL) methods have gained a lot of attention from industry and academia due to their superior performance in various applications, including CV, natural language processing, transportation, healthcare, finance, visual recognition, cybersecurity, etc. [10,16]. In what has traditionally been a field of simple metrics and labor-intensive video review, the transportation industry and related academia have explored AI approaches for improved DMSs by combining two or three of these approaches via DL and data fusion [11,12,17]. The AI methods provide comprehensive datasets to better understand how drivers react under different environmental factors, including both the road scene and the psychological state of the driver, to provide recommendations for optimizing DMS output towards the identification of at-risk driver behaviors and conditions using suitable system devices and signals.

Moreover, AI tools have been utilized to analyze Safety Critical Events (SCEs) such as crash events, near-crash events, or driver errors. These tools leverage camera data from various sources, including traffic operations and onboard DMSs, to understand the causal factors behind crashes. Using this information, practitioners can recommend changes in driver training, infrastructure or road design, or other countermeasures to minimize crash risk. The substantial potential of these tools lies in their ability to reduce the frequency and severity of traffic accidents, subsequently curbing associated fatalities, injuries, property losses, traffic congestion, and expediting efficient rescue operations. As such, AI models have been utilized to predict hazardous driving events from DMSs [18] or SCEs from naturalistic driving data [19]. DL models have also been applied to integrate drivers’ visual characteristics into the collision warning system to discover potential dangers earlier and shorten reaction time [20]. Hussain et al. [21] used AI and CV techniques to forecast crash risks at signalized intersections for the next 30–35 min with reasonable accuracy. Tian et al. [22] leveraged DL and CV to automatically detect vehicle accidents to shorten the response time of rescue agencies and vehicles around accidents.

**Figure 1:**
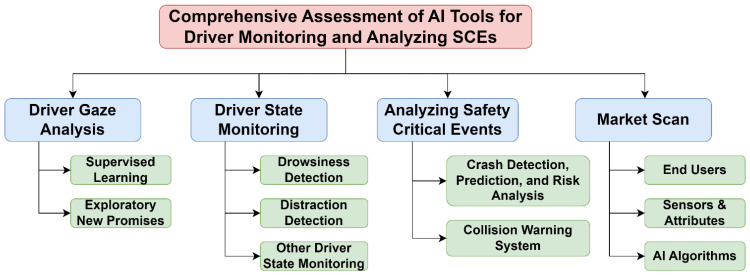
Comprehensive assessment of AI tools for driver monitoring and analyzing SCEs.

To better understand the state-of-the-art practices and potential directions for AI tools for driver monitoring and analyzing SCEs in vehicles, this work marks the inaugural attempt to consolidate AI-related tools for driver behavior monitoring and analyzing SCEs from academic and industry perspectives. It provides a robust foundation with existing knowledge summarized, gaps identified, and future directions recommended to researchers and practitioners interested in leveraging AI tools to enhance driving safety for vehicles, including trucks and buses, via minimizing driver errors and avoiding SCEs. As shown in Figure 1, this study comprehensively reviewed online publications and conducted a market scan involving advanced CV, ML, and DL via four avenues:

- Driver gaze analysis. Summarizes previous works via supervised ML/DL and exploratory new promises for driver gaze tracking, classification, or estimation in terms of devices, datasets, methodologies, and results.
- Driver state monitoring. Includes methods, devices, features, data, and results of drowsiness detection, distraction detection, and others (such as emotion, drunk driving, or other dangerous driving behaviors) via CV, ML, and DL methods.
- SCE analysis. One of the direct outcomes of analyzing SCEs is to understand how driver behaviors relate to overall crash risk. This section reviews the state-of-the-art practice for crash detection, prediction, and risk analysis, and the development of collision warning systems and their impact on drivers’ behavior.
- Market scan. Identifies commercially available DMSs in vehicles that use AI and ML, and summarizes sensor types, industry trends, and gaps in current DMS technologies.

## 2. Driver Gaze Analysis

Human gaze analysis is a process to estimate and track a person’s 3D line of sight (i.e., where a person is looking) [23,24]. Gaze analysis has been an interesting topic in CV across various research areas, such as human–computer interaction [25], head-mounted devices [26], driver behavior monitoring [27], and healthcare [28]. Also, driver gaze analysis has been part of DMS to monitor driver’s attention, focus, and visual engagement throughout various driving scenarios to identify and mitigate risky driver behavior. Gaze analysis was traditionally performed via color, shape, appearance, and certain geometrical heuristics of eye or facial images in CV [24,29,30]. The DL approach has been mainstreamed for gaze analysis since 2015 due to its superior performance. In the era of automated driving, driver gaze analysis is an important topic in Advanced Driver Assistance Systems (ADAS) to monitor a driver’s awareness to minimize crash probabilities and improve roadway safety [27,31,32]. Compared with other applications, driver gaze analysis using camera data is challenging due to its diverse and dynamic data collection environment, which includes such elements as driver appearance (presence of sunglasses, hats, occlusion due to hair, hands), rapid change in ambient light, vehicle vibration causing image blur, and requirements for real-time processing [30,31]. This section presents a comprehensive review of ML or DL-based driver gaze analysis using images or videos for DMS. It also covers some of the latest gaze analysis studies from other applications to explore future directions of driver gaze analysis in DMS.

### 2.1. Supervised Learning

Many studies have been conducted for real-time driver gaze analysis in CV via traditional supervised ML methods that are trained using labeled data for DMS. Fridman et al. [31] collected more than 1.8 million grayscale image frames from 50 subjects via video cameras to classify driver gaze into six regions (road, center stack, instrument cluster, rearview mirror, left, and right) with an average accuracy of 91.4%. The algorithm used a pipeline of (a) face detection via Histogram of Oriented Gradients (HOG) combined with a linear Support Vector Machine (SVM) classifier, (b) face alignment via a 56-point facial landmark, (c) feature extraction, normalization, and selection, and (d) classification via random forest and decision pruning. Figure 1 in [31] shows example images of correct and incorrect predictions of gaze regions via facial landmarks and random forest. In another study, a low-cost Charge-Coupled Device (CCD) camera was placed on top of the steering wheel column to capture images of driver’s face with the assistance of an infrared (IR) illuminator for nighttime operation for gaze tracking [33]. The facial features detected via pyramidal Gabor wavelets and the head pose estimation from a normalized Singular Value Decomposition were applied for gaze estimation via a hierarchical generalized regression neural network to achieve an accuracy of 92%.

Moreover, Wang et al. [34] used a Red Green Blue-Depth (RGB-D) camera that provides both color (RGB) and depth data to perform appearance-based estimation for nine gaze zones. A total of 50,000 RGB data and depth data units from a single driver in natural driving environment were prepared. The head pose from a cascaded nearest neighbor query and the gaze angle prediction from the local feature regression were calculated to determine the gaze zones. Recently, Shan et al. [35] collected 90,791 photos with 20 drivers involved, including 9 with glasses, to estimate 10 gaze regions in a real car. The facial landmarks were obtained from a regression tree set for head posture acquisition via a Pose from Orthographic and Scaling with Iterations (POSIT) algorithm and pupil position and eye feature extraction. The improved random forest combined the head and eye features to classify gaze regions with an accuracy of 94.12%. Ledezma et al. [32] used a Microsoft Kinect v2.0 sensor (RGB and IR cameras) for gaze tracking in a driving simulation environment with clear light conditions. The research used 27,000 frames of three participants for extraction of the eye Region-Of-Interest (ROI) and estimation of pupil center coordinates via an Emgu CV library, achieving promising results with hit ratios between 96.37% and 81.84%.

**Figure 2:**
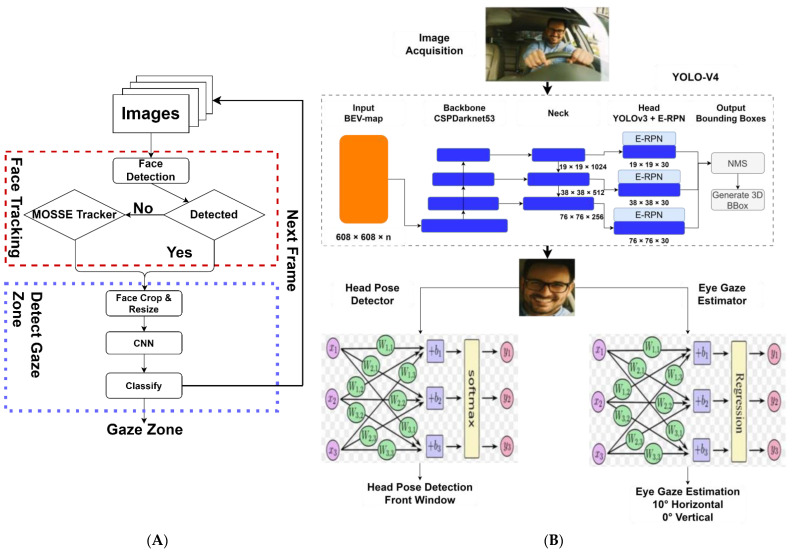
Driver gaze estimation via DL models. (**A**) CNN (adapted from Ref. [36]). (**B**) YOLO-V4 and CNN (adapted from Ref. [27]).

Furthermore, studies using supervised DL models for driver gaze analysis via images or videos achieved outstanding performance compared with the traditional ML method. For example, Choi et al. [36] combined the Haar feature and the Minimizing the Output Sum of Squared Error tracker for face tracking, and implemented a Convolutional Neural Network (CNN) to classify detected face images into nine gaze zones with 95% detection rate via 35,900 images of four drivers from a CCD camera, as illustrated in Figure 2A. Naqvi et al. [37] utilized a Near-Infrared (NIR) camera and an illuminator of six NIR LEDs to capture a driver’s frontal view of 20 drivers, including 3 wearing glasses. The study developed three CNN models of face, left, and right eye ROI images to classify 17 driver gaze zones, achieving an average detection rate of 92.8% for Strictly Correct Estimation Rate (SCER) and 99.6% for Loosely Correct Estimation Rate (LCER). Vora et al. [38] prepared 47,515 images of 11 drivers from different time via a RGB camera mounted near the rearview mirror for seven gaze zone classifications via four separate CNNs (AlexNet, VGG16, ResNet50 and SqueezeNet). The results showed that the fine-tuned SqueezeNet achieved 95.18% accuracy with images of upper half of the driver’s face without requiring any ground truth annotations of the eye or the face, thereby completely removing the need for face detection.

Then, Rangesh et al. [39] built Gaze Preserving CycleGAN (GPCycleGAN) for eyeglass removal and driver’s gaze classification via SqueezeNet for seven gaze zones (eyes closed/lap, forward, left mirror, speedometer, radio, rearview, and right mirror). An IR camera installed next to the rearview mirror was used to detect 336,177 images collected under different lighting conditions (daytime, nighttime, and harsh lighting) from 13 subjects wearing various eyeglasses. The model detected landmarks via OpenPose, cropped eye images, and achieved an accuracy of 80.49% for gaze estimation. Shah et al. [27] proposed a real-time system for estimating head pose direction via YOLO-V4 and InceptionResNet-v2, and eye gaze tracking horizontally and vertically via CNN regression, as illustrated in Figure 2B. The model was trained based on a custom dataset containing 83,662 images for seven classes of head poses and 135,409 images for 10 eye gaze angles that were collected from 30 participating individuals using a high-resolution camera.

Some efforts attempted to combine drivers’ facial information with other features, such as vehicle cabin environment, road, and vehicle signals, for better driver gaze estimation. For instance, Stappen et al. [40] combined the driver’s face with images from the surrounding environment (for example, the vehicle cabin environment) for gaze estimation of nine zones via revised InceptionResNetV2. The 50,000 images were collected from 247 male and 91 female subjects with most being between 18 and 35 years old via a Microsoft LifeCam RGB camera that was positioned frontally to the test person. The proposed method, based on the full image (environment and face) or the full set of features (facial and Go-CaRD features; as illustrated in Figure 2 in [40]), outperformed other DL models, such as InceptionV3, ResNet50, VGG16, and VGG19. Recently, Kasahara et al. [41] presented a new dataset, called “Look Both Ways”, which contains synchronized video of both driver faces and the forward road scene for gaze estimation and road scene saliency. The Look Both Ways dataset contains 123,297 synchronized driver face and stereo scene images with ground truth 3D gaze, which were collected from 6.8 h of free driving on public roads by 28 drivers. The proposed method used self-supervised learning to consider the gaze estimation from facial images via the ETH XGaze model, and saliency estimation from visual scene saliency via the Unisal (MNetV2-RNN-Decoder).

### 2.2. Exploratory New Promises

**Figure 3:**
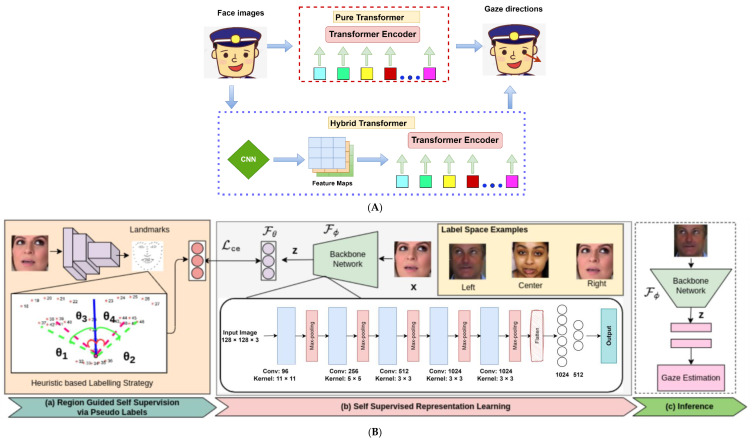
Exploratory new promises for gaze estimation. (**A**) Gaze direction estimation using transformer (adapted from Ref. [42]). (**B**) RAZE framework (adapted from Ref. [47]).

As transportation engineers are improving driver gaze analysis, some avenues have also been explored to improve gaze analysis in other fields using advanced methodologies. These new advancements shed light on future directions for better real-time gaze analysis of vehicle drivers for DMS. For instance, Cheng and Lu [42] employed a pure transformer and hybrid transformer to estimate gaze directions from images, as shown in Figure 3A. Specifically, the pure transformer estimated gaze directions from patches of face images, whereas the hybrid transformer applied ResNet-18 to extract feature maps of face images and used transformer for gaze direction estimation. The hybrid transformer achieved superior performance over the pure transformer in all evaluation datasets with fewer parameters. A novel multi-resolution fusion transformer model was developed to efficiently estimate gaze based on multi-resolution feature maps with global and local information from the neural architecture search for real-time applications [43]. These recently developed models showed promising performance and should be explored for real-time driver gaze analysis in ADAS.

Furthermore, gaze analysis using supervised methods always requires large scale annotated data, which is expensive and time consuming to acquire [24,25,29,44,45]. Therefore, some studies applied unsupervised or limited supervision methods for gaze analysis without gaze annotations on images or videos. For example, Yu and Odobez [46] presented an unsupervised representation learning for gaze estimation without annotations of 3D gaze data. The model contains three major parts: (1) a network based on ResNet blocks to extract the gaze representations from the input images and compute the representation difference, (2) an alignment sub-network to predict the motion parameters (translation and relative scale) between an input image and a target output, and (3) a trained encoder-decoder network to predict a warping field which warps the input using a grid sampling operation and synthesizes a gaze redirection output. Next, Dubey et al. [47] proposed RAZE to learn gaze representation via auxiliary supervision to overcome the requirement of large scale annotated data, as shown in Figure 3B. RAZE first performs pseudo labelling of the detected faces based on facial landmarks, then maps input image to the label space via a backbone network aka “Ize-Net”. Unfortunately, studies via unsupervised DL methods for detailed driver gaze analysis were not yet available, based on the extensive literature review.

Lastly, Virtual Reality (VR) technology can create, manipulate, and control the environment that an individual is immersed within, with situations ranging from simple lab environments to much more complex real-world setups [48]. Therefore, VR devices have been applied for gaze analysis in some studies to minimize the complexity of hardware configuration and cost for data collection. For example, Blattgerste et al. [49] showed that eye-gaze based VR head-mounted systems outperformed head-gaze based systems for aiming and dwell-time or clicking for triggering the selection in terms of speed, task load, required head movement, and user preference. Hu et al. [50] developed the SGaze (as illustrated in Figure 1 in [50]) to predict real-time gaze position in an immersive VR system using head movements and other factors. Particularly, the eye–head coordination model was developed to predict gaze position based on a dataset that was recorded from 60 participants (35 male, 25 female, ages 18–36) via eye tracker hardware and different VR scenes (city, desert, forest, etc.) under various lighting conditions. Accordingly, if VR is applied for driver gaze analysis, it would be cost effective to prepare a large dataset with high-resolution images under various circumstances without requiring participants to drive vehicles on different roads for thousands of miles under varying time and weather conditions (e.g., day, night, sunny, cloudy, storm, snow, etc.). Combining that large high-resolution dataset with advanced DL methods (transformers, unsupervised learning, etc.) should benefit and improve the performance of real-time driver gaze analysis.

Particularly, Table 1 summarizes the method, camera, database, input data, features, image resolution, accuracy, and number of gaze zones of selected studies for driver gaze analysis via CV. The intention of Table 1 is not to engage in direct result comparisons, but rather to present an overview of their respective models, datasets, input features, and training outcomes of previous work for driver gaze analysis via AI tools. The major findings and gaps from these studies are as follows:

- Compared with traditional CV techniques, DL methods (CNN, VGG, ResNet, GAN, etc.) improved the performance of image-based driver gaze analysis in many studies. However, other recent DL models, such as transformer or unsupervised learning, should be explored to improve the accuracy of driver gaze analysis.
- As shown in Table 1, there are some limitations of current datasets for driver’s gaze analysis. Limitations may include, for example: low image resolution; dataset not large enough to have adequate training samples for all gaze zones; and limited data collection during abnormal weather (rain, snow, wind, etc.). More high-resolution images of drivers’ faces or eyes under different scenarios (weather, traffic, roads, illumination, etc.) are desired in the future for model training.
- As shown in Table 1, the number of gaze zones among these studies are not consistent; they range from 5 to 17. Determining the critical driver gaze zones is crucial to maintain safety during driving. Accordingly, a robust algorithm to monitor the identified critical gaze zones of drivers can be developed for better DMS or ADAS.
- In addition to driver’s facial images, more data sources should be included for a comprehensive driver’s gaze analysis during naturalistic driving situations. For instance, images or videos of roads should be collected to monitor road condition, traffic flow, and understand the corresponding driver behavior or gaze movement.
- Current studies mostly focus on classifying drivers’ gaze zones via images or video. A real-time prediction of driver’s gaze during driving among those zones via AI and CV should benefit DMS and ADAS.
- Gaze analysis of truck or bus drivers is absent based on the literature review. Given the distinct visibility challenges posed by the larger and higher cabs of trucks and buses compared to passenger vehicles, there is a need to investigate the critical gaze zones for truck or bus drivers to ensure safe driving practices.

## 3. Driver State Monitoring

In addition to driver gaze analysis, extensive studies have been conducted to perform driver state monitoring via various sensors and techniques for driver monitoring to identify and mitigate risky driver behavior. Driver state is closely related to alertness, reaction time, and risky driving behaviors, which may lead to SCEs during driving. This section summarizes how AI tools benefits driver state monitoring, including drowsiness detection, distraction detection, and others (e.g., emotional analysis, drunk driving, or other dangerous driving behaviors).

### 3.1. Driver Drowsiness Detection

Driver drowsiness impacts a driver’s alertness and response time and increases the probability of vehicle accidents; drowsy driving contributes to about 20% of all car crashes [12]. Therefore, it is critical to monitor a driver’s level of drowsiness to alert drivers when necessary to minimize roadway accidents. Usually, driver fatigue or drowsiness detection may be accompanied by such physiological variables such as eye movement, facial expression, heart and breathing rate, and brain activity [8]. This section summarizes recent studies of driver drowsiness detection via CV and ML methods.

**Figure 4:**
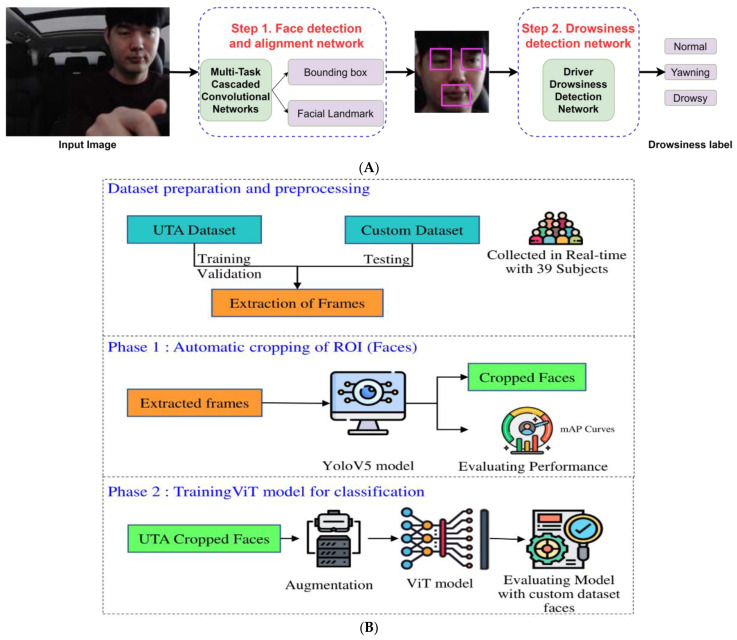
Drowsiness detection via DL models. (**A**) Drowsiness detection via CNN (adapted from Ref. [53]). (**B**) Driver drowsiness detection via YOLO-V5 and Vision Transformers (adapted with permission from Ref. [57]).

For instance, Vural et al. [51] predicted driver drowsiness via ML methods (Adaboost classifier and multinomial ridge regression [MLR]) from driving simulation videos of 100 university students. Combining head motion and facial actions, including blinking and yawning motions, Adaboost obtained 92% accuracy and MLR obtained 94% accuracy when predicting alert or non-alert drivers. Later, 5700 thermal images from 19 subjects were applied to classify fatigued drivers via AlexNet for feature extraction from facial images and SVM to classify fatigue state and resting state with an accuracy of 80% [52]. To achieve real-time drowsiness detection via an embedded system, Reddy et al. [53] collected 70,000 images of 33 subjects from diverse ethnic groups and gender, including 11 people with glasses, and developed a compressed CNN model using the cropped images of the left eye and the mouth. As shown in Figure 4A, the proposed model consisted of two parts: the Multi-Task Cascaded CNN for the face detection and alignment task, and the Driver Drowsiness Detection Network for detecting driver drowsiness. The model achieved an accuracy of 89.5% on 3-class classification (normal, yawning, and drowsy) with a speed of 14.9 frames per second using a Jetson TK1. Further, Revelo et al. [54] collected 2400 images from 8 persons via IR camera for drowsiness detection. Classification of open and closed eye was performed via two methods: (1) using landmarks of the eye image to determine the maximum and minimum of horizontal and vertical edges of the eye, and (2) applying a multilayer perceptron (MLP) neural network to classify pixels of eye images. The accuracies were 84% for the first method and 97% for the second method. Hashemi et al. [55] developed a CNN model for drowsiness detection via 4185 cropped eye images of four persons, and achieved 96.39% accuracy on the prepared testing images.

More recently, Draz et al. [56] tested a Raspberry Pi-4 with 8 GB RAM using a Logitech HD720 webcam to track a driver’s face and eyes for detecting drowsiness in real-time with an average accuracy of 97.3%. The method applied the Dlib face detector to segment eyes from the face image, and calculated the Eye Aspect Ratio of the driver’s eyes to decide if the driver was in a drowsy state. Das et al. [12] developed a driving simulation system with four physiological sensors, three RGB cameras, an NIR camera, and two thermal cameras to detect drivers’ drowsiness and distraction. The results showed that the physiological modality provided the best performance of an 84% F1-score for a drowsiness label. Krishna et al. [57] prepared 9180 images from The University of Texas at Arlington Real-Life Drowsiness Dataset with 36 subjects and 1246 customer images with 39 subjects for driver drowsiness detection. The proposed method achieved 95.5% accuracy via YOLO-V5 to detect driver’s face and vision transformers to classify drivers as drowsy or alert, as shown in Figure 4B. Sharak et al. [58] compared four contact-based methods (physiological sensor) against three noncontact-based methods (RGB camera, NIR camera, and thermal camera) for driver drowsiness detection using multimodal dataset from 45 subjects (gender: 30 male and 15 female, ages: 20–33, ethnic groups: 6 White/Caucasian and 24 Asian/Middle Eastern). The results indicated that the NIR and visual cameras showed better performance for noncontact-based drowsiness monitoring, and were cheaper and easier for installation. Alameen and Alhothali [59] developed a model with 3DCNN and Long Short-Term Memory (LSTM) integrated to understand the deep long-term spatiotemporal correlation for driver drowsiness detection via frontal and side facial images, and achieved an accuracy of 96% for YawDD with 29 subjects, 93% for Side-3MDAD, and 90% for Front-3MDAD which contains 50 participants from diverse ages, gender, and body sizes.

### 3.2. Driver Distraction Detection

NHTSA defines distracted driving as “any activity that diverts attention from driving, including talking or texting on your phone, eating and drinking, talking to people in your vehicle, fiddling with the stereo, entertainment or navigation system—anything that takes your attention away from the task of safe driving” [60]. The number of fatalities in distraction-affected crashes is much higher than those involving a drowsy driver based on NHTSA’s statistics for recent years [61]. Furthermore, Level 2 automation requires drivers to take over control of vehicles. Research shows that drivers are often distracted and engaged in other secondary behavior in highly automated vehicles. Driver secondary behavior includes eating, drinking, the act of picking something up, tuning the radio, or the use of cell phone and other technologies [62]. Therefore, understanding drivers’ exact posture, attentiveness, and readiness to takeover is important for safe operations of the vehicle. This section mainly discusses methods to automatically identify driver distraction via CV and ML methods.

**Figure 5:**
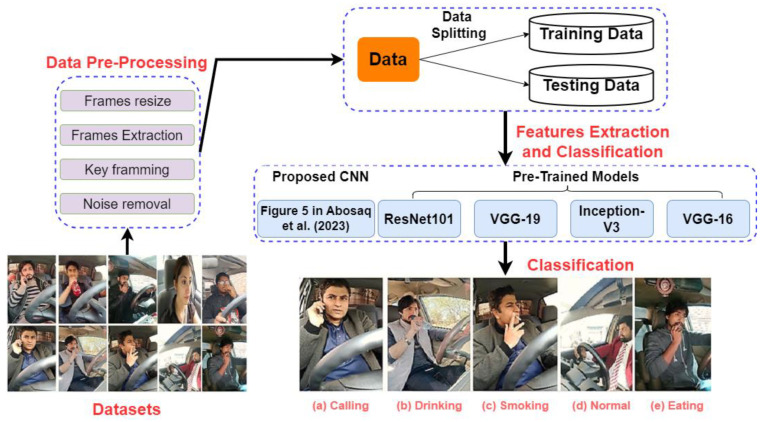
Driver unusual behavior detection via CNN (adapted from Ref. [65]).

Zhao et al. [63] captured drivers’ side images of 20 participants (10 male and 10 female) via a video camera to develop the southeast university (SEU) dataset for recognizing four driving postures (grasping the steel wheel, operating the shift lever, eating, and talking on a cellular phone) with 88% accuracy by using contourlet transform for feature extraction and random forests for posture classification. Later, Yan et al. [64] used the SEU dataset to classify six driver behaviors (responding to phone call, eating while driving, operating the shift gear, correct driving position with hands on wheel, playing with phone while driving, and driving while smoking) via a Gaussian Mixture Model to extract skin-like regions and using CNN to generate action labels on videos, achieving a mean average precision (mAP) of 97.97%. Abosaq et al. [65] proposed a customized CNN model (Figure 5) to recognize normal and abnormal driver actions (including driver smoking, driver eating, driver drinking, driver calling, and driver normal) from driver videos, and achieved 95% accuracy on the prepared testing dataset. Yang et al. [66] investigated the impacts of feature selection on driver cognitive distraction detection and validation in real-world non-automated and Level 2 automated driving scenarios. A Mobileye sensor recorded vehicle performance while two Logitech webcams and a forward-facing camera collected video data of 24 drivers (12 males and 12 females with ages 22–68) and roadway. The results concluded that combining transformed eye (e.g., gaze, blink, and pupil), head, and vehicle-control features with glance features can enhance cognitive distraction classification performance. Hou et al. [67] combined Mobilenet and a single shot multi-box detector (Mobilenet-SSD) to detect mobile phone usage while driving from 6796 driving images, and achieved an accuracy of 99% on the prepared testing images.

### 3.3. Other Driver State Monitoring

**Figure 6:**
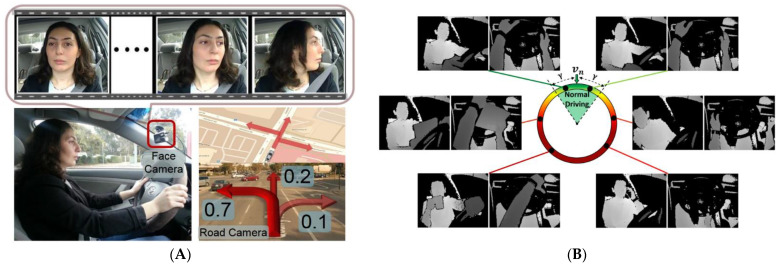

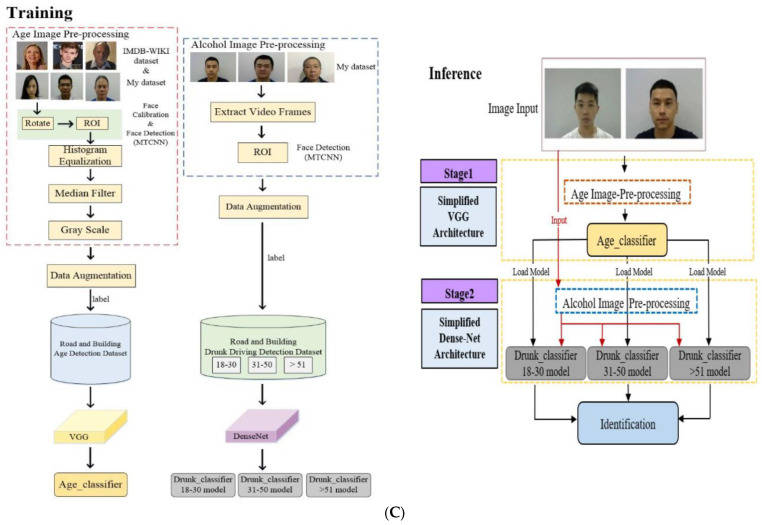
Driver state monitoring from other aspects. (**A**) Multiple data sources for maneuver anticipation via LSTM (adapted with permission from Ref. [68]). (**B**) Driver anomaly detection via multi-modal (depth and infrared) and multi-view (front and top) images (adapted with permission from Ref. [71]). (**C**) Drunk driving detection system via two-stage neural network (adapted with permission from Ref. [70]).

In addition to driver drowsiness and distraction detection, many studies explored other driver state monitoring via video/images only or using a multimodal approach. For example, Jain et al. [68] developed a vehicular sensor-rich platform with cameras, GNSS, and a computing device to capture the driving context from both inside and outside of the car for maneuver anticipation via LSTM, as shown in Figure 6A. The prepared dataset, Brain4Cars, had 2 million video frames from 1180 miles of highways from 10 drivers with diverse landscapes, and the proposed model achieved 90.5% accuracy and 87.4% recall, anticipating drivers’ maneuvers (lane change, turns, and all other maneuvers) 3.5 s before they occurred in real-time. Also, some studies focused on the relationship between drivers’ emotions and driving circumstances. For instance, Balali et al. [11] had a naturalistic driving setup consisting of videos recording both the driver and the road via a Z-Edge S3 Dual Dashcam, heart rate data, and data from the car’s Controller Area Network. Results suggested that weather conditions and road types may significantly change driver emotions and driving behavior. Furthermore, unsupervised learning of naturalistic driving data was performed to determine patterns of driving behaviors, driver’s heart rates, and gaze entropy [17]. The IMU, smart watches, and in-cabin and outdoor facing cameras were used to detect a driver’s state. The results indicated that drivers had high heart rates during harsh brakes, when accelerating, and during curved driving, whereas low heart rates and low gaze entropy patterns were seen during free-flow driving.

Recently, some studies have explored the detection of drunk driving via different sensors or methods. Sharma and Sood [69] employed an alcohol sensor and air pressure sensor for sobriety checks, and ML algorithms for drivers’ drowsiness detection via camera. Chang et al. [70] explored drunk driving detection via facial images and breath-alcohol tester from 124 subjects (ages 18–70) using simplified VGG and Dense-Net: VGG classified the age range of the subject while Dense-Net identified the facial features of drunk driving for alcohol test identification, as shown in Figure 6C. The model achieved an accuracy of 87.44% and the results showed that (1) the ears, chin, forehead, neck, cheek, and other facial parts of subjects’ images are good characteristic areas for alcohol tests, and (2) age affects the identification results in the alcohol test.

Some researchers also applied CV to identify anomalies or dangerous driving behaviors. For instance, the Driver Anomaly Detection dataset, which is comprised of multi-modal (depth and infrared) and multi-view (front and top) images of 31 drivers obtained from a driving simulator, was examined to investigate driver anomaly detection and classification, as shown in Figure 6B [71]. The analysis employed MobileNetV2 and achieved an impressive 0.9673 Area Under the Curve (AUC) on the receiver operating characteristic curve. Xiang et al. [72] used a cloud model and Elman neural network to predict dangerous driving behavior, including slow speeding, urgent acceleration, slow speed reduction, general slowdown, and sharp slowdown, based on vehicle motion state estimation and passengers’ subjective feeling scores.

Lastly, Table 2 summarizes the application, methods, device, feature, data, number of classes, and results of selected studies for driver state monitoring via AI tools. Again, the intention of Table 2 is not to engage in direct result comparisons, but rather to present an overview of their respective models, datasets, input features, and training outcomes of previous work for driver state monitoring via AI tools. The major findings and gaps from these studies are as follows:

- Driver state monitoring encompasses a wide range of facets, such as identifying drowsiness, detecting distractions, predicting maneuvers, monitoring driver emotions, detecting drunk driving, and identifying driver anomalies.
- DL methods have significantly enhanced the effectiveness of image-based driver state monitoring in various aspects, surpassing traditional CV techniques, just as they have done with driver gaze analysis.
- Noncontact-based drowsiness monitoring using CV and DL methods showed better performance than contact-based methods and were cheaper and easier for installation.
- The future of driver state monitoring is poised to leverage advanced DL models, facilitating the integration of multi-modal (RGB, depth, or IR) and multi-view (front, top, or side) images. This approach will pave the way for more comprehensive and robust driver state monitoring systems in real-time.
- State monitoring of truck or bus drivers is limited, based on the literature review.

## 4. Analyzing Safety Critical Events

Beyond driver monitoring to identify and mitigate risky driver behavior, AI tools have been applied to analyze SCEs and implement necessary actions to prevent accidents from happening. Furthermore, one goal of self-driving cars is for them to learn and anticipate the behavior of other human-driven vehicles or highly automated vehicles to avoid accidents. However, analyzing SCEs is addressed less often than anticipating specific maneuvers such as lane changes or turns because predicting traffic accidents poses significant challenges due to their diverse nature and the suddenness with which they typically occur [73]. Recently, some efforts have been conducted to predict crash risk or prevent future crashes via various sensors and methodologies. This section presents a summary of ML- or DL-based studies of analyzing SCEs using CV, including crash detection, prediction, risk analysis, and collision warning systems.

### 4.1. Crash Detection, Prediction, and Risk Analysis

Traditionally, researchers have focused on ML- or DL-based crash detection or prediction on freeways, urban arterials, or intersections to manage roadway safety proactively using various datasets, such as those containing traffic data, signal timing data, weather data, roadway attributes, and/or driver behavior. For instance, Li [74] applied LSTM-CNN to predict real-time crash risk at arterials via traffic data, signal timing data, and weather data in Orlando, FL, and achieved better performance than five other benchmark models in terms of AUC, sensitivity, and false alarm rate. Recently, some studies have tried to predict crashes earlier in real-time from the ego-vehicle’s perspective using different sensors and methodologies to reduce crash probabilities. For example, Chan et al. [73] proposed a Dynamic-Spatial-Attention RNN (DSA-RNN) model to anticipate accidents in 678 dashcam videos from six major cities in Taiwan. The model fusing VGG appearance and improved dense trajectory motion features achieved accident anticipation about 2 s before an accident occurred with 80% recall and 56.14% precision. Typical accident anticipation examples in dashcam videos via DSA-RNN can be found in Figure 5 in [73]. Later, Suzuki et al. [75] developed a quasi-recurrent neural network using Adaptive Loss for Early Anticipation (AdaLEA) for traffic accident anticipation from the 4594 self-annotated Near-miss Incident traffic videos. The model achieved better performance than conventional models in terms of mAP (62.1%) and average time-to-collision (ATTC; 3.65 s) for risk anticipation.

**Figure 7:**
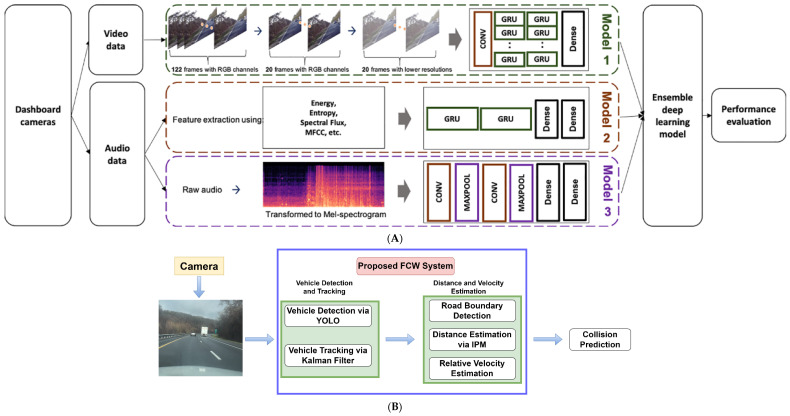
Example studies for analyzing SCEs. (**A**) Car crash detection via video and audio data (adapted with permission from Ref. [76]). (**B**) Forward collision warning system via monocular vision (adapted from Ref. [86]).

Furthermore, Choi et al. [76] combined gated recurrent unit (GRU) and CNN for a car crash detection system using video and audio data from dashboard cameras to assist an emergency road call service that recognizes traffic accidents automatically. As illustrated in Figure 7A, the model has three main components for car crash detection: (1) crash detection from videos using CNN and GRU, (2) crash detection from audio features via GRU and audio spectrogram via CNN, and (3) a weighted average ensemble model to combine the three different classifiers for final crash detection. The model was trained and tested with 500 video clips, and the results demonstrate that the incorporation of multiple data sources outperforms the use of a single data type, leading to improved performance of more than 89% with AUC. Also, Shi et al. [77] analyzed the longitudinal, lateral, and vertical acceleration data from 1820 crashes, 6848 near-crashes, and 59,997 normal driving events in the SHRP 2 naturalistic driving study to perform real-time driving risk assessment via CNN and GRU with Extreme Gradient Boosting (XGBoost). The model achieved an overall accuracy of 97.5% to classify crash, near-crash, and normal driving segments.

Some studies have proposed crash risk assessment via driving scene analysis using CV. For example, Karim et al. [78] developed a driving scene analysis system in support of crash risk assessment and crash prevention, as shown in Figure 1 in [78]. A total of 15,900, 6400, 7900, and 7400 images from dashcams were prepared to classify crash likelihood (pre-crash, crash, no-crash), road function (arterial, collector, interstate, local), weather (rainy, snowy, clear, overcast, foggy), and time of day (daytime, night, dawn/dusk) via Multi_Net, which included DeepLabv3 and YOLOv3 for image classification and segmentation. The findings revealed that the analysis of driving scenes through vision sensors can equip automated vehicles or human drivers with situational awareness, enabling them to identify potential crash risks within the surrounding traffic. To assist earlier crash prediction using CV, Li et al. [79] proposed scenario-wise, spatio-temporal attention guidance to estimate the relevance of detected objects from images or videos to specific fatal crash risks. The results indicated that combining attention guidance and CV for driving scene analysis had the potential to enhance drivers’ awareness regarding objects that demand greater attention to enhance safety.

Moreover, in certain studies, crash risk prediction has been conducted by incorporating surrogate safety measures. For example, Li et al. [80] introduced an attention-based LSTM model for lane change behavior prediction considering the current and historical trajectory data of the vehicle, and further verified the effectiveness of a crash risk prediction model during lane change based on Time to Collision (TTC) in an example study. In another study, Yao et al. [81] combined a shockwave module with features extracted from CNN and LSTM models as a Physics-informed Multi-step real-time conflict-based vehicle safety prediction model using historical vehicle trajectory data to make predictions of conflict-based vehicle safety indicators. The safe stopping distance difference between two consecutive vehicles was calculated from the HIGHSIM data, and the vehicle safety prediction model was compared to three benchmark models (LSTM-CNN, ANN-state, and Autoregressive Integrated Moving Average Model) to demonstrate its superior performance when predicting risky or safe driving.

Lastly, certain researchers have delved into alternative perspectives of ML-based crash risk analysis by utilizing diverse techniques and data sources. To solve the data scarcity problem of collecting and labeling real (near) collisions, Schoonbeek et al. [82] trained a perception module to predict optical flow and object detection from a sequence of RGB camera images, and proposed RiskNet to classify individual frames of a front-facing camera as safe or unsafe. The RiskNet was trained on a simulated collision dataset (58,904 safe and 7788 unsafe frames) and tested on real-world collision dataset (3604 safe and 1008 unsafe frames) with an accuracy of 91.8% and F1-score of 0.92. In another study, Zheng et al. [83] used naturalistic driving data from The 100-Car Naturalistic Driving Study to classify distraction risk levels via driver’s gaze or secondary driving tasks. They combined distraction risk levels, road environment factors, and driver characteristics to predict influencing factors on accident occurrence via random forest, AdaBoost, and XGBoost. The results indicated that drivers’ gaze is more related to their distraction levels, and that XGBoost had superior performance over other methods to predict accident occurrences. Zhang et al. [84] proposed a proactive crash risk prediction framework for lane-changing behavior incorporating individual driving styles using the trajectory data in the highD dataset. The framework implemented a dynamic clustering process to classify driving styles and used the Light Gradient Boosting Machine to predict lane-changing risk for cautious, normal, and aggressive drivers. The results indicate aggressive drivers may have higher lane-changing risk and suggest that ADAS should contain a lane-change warning system to ensure driving safety. Loo et al. [85] used Negative Binomial, XGBoosting, and random forest models to verify the effects of five risk factors (pedestrian volume, pedestrian crowding, jaywalking, missing railing, and sharp turns) for bus-pedestrian crashes. The bus dashcam videos of 12,679 bus-related crashes in Hong Kong were processed for pedestrian tracking, generating the jaywalking index, and detecting sidewalk railings via Fast R-CNN, Mask R-CNN, and segmentation, individually. The study findings emphasized the significance of pedestrian exposure, jaywalking, crowding, and sidewalk railings as crucial elements to be considered when addressing bus–pedestrian crashes.

### 4.2. Collision Warning System

In addition to crash detection, prediction, and risk analysis, substantial resources have been invested in developing collision warning systems to mitigate crash risks and enhance the safety of roadways. For example, a real-time collision avoidance system was developed by fusing Light Direction and Ranging (LiDAR) and camera data to detect passive beacons to stop the vehicle from entering a restricted space [87]. The results showed that fusion helps to obtain more accurate position and label information in various prototyping scenarios. Venkateswaran et al. [86] developed a monocular vision-based forward collision warning system (as shown in Figure 7B), which included three main components: (1) detecting on-road vehicles via a pre-trained YOLO, (2) assigning a unique ID for detected vehicles using a Hungarian algorithm and tracking detected vehicles via Kalman filter, and (3) calculating the distance between the detected vehicle and the ego-vehicle. By testing on different datasets, the system achieved more than 0.85 precision for vehicle detection and less than 9.14 RMSE for vehicle tracking. Also, Rill and Faragó [88] proposed a DL based forward collision avoidance system which estimated TTC based on a monocular vision algorithm. They used a spherical camera and a pair of smart glasses to collect more than 10 h of driving videos, developed a CNN model for monocular depth estimation and the pre-trained YOLOv3 for object detection, and estimated the speed of the ego-vehicle and TTC for ADAS to react before collision. Gómez-Huélamo et al. [89] presented a real-time and power-efficient 3D Multi-Object Detection and Tracking method via merging obstacles from LiDAR and features from camera to track 360° surrounding objects for forecasting trajectories and preventing collisions for the ego-vehicle.

Furthermore, animal detection and collision avoidance systems have been investigated by some researchers to improve vehicle safety. For instance, Sharma and Shah [90] trained an animal detection algorithm using HOG and a cascade classifier based on 2200 images with different animals on highways under different driving speeds. The system achieved an accuracy of 82.5% when detecting animals and could alert the driver under speeds of 35 km/h to prevent a collision. In another study, Gupta et al. [91] developed an animal avoidance system for automated vehicles using dashcam and multiple models, including animal detection via Mask R-CNN, lane detection, animal direction, and vicinity tracking via a centroid tracking algorithm. The framework was able to detect and track animals to determine if there was a collision possibility for vehicles with a decent accuracy: 79.47% and 81.09% accuracy for detecting cows and dogs, 84.18% for accident detection ratio, and 0.026% for false alarm rate. Saxena et al. [92] created a dataset with 31,774 images of 25 animal categories and applied single shot multibox detector (SSD) and Faster R-CNN for animal detection to reduce animal–vehicle collision. The SSD achieved 80.5% mAP at faster speed (100 fps) while the Faster R-CNN achieved 82.11% mAP at slower speed (10 fps) for animal detection on the testing dataset. Mowen et al. [93] used a thermal camera to collect 111 thermal images of animals during nocturnal hours in Texas to classify animal poses for assessing the risk posed by an animal to a passing automobile. The developed CNN model achieved an average accuracy of 82% to classify animal poses into lying down, facing toward automobile, and facing away from automobile to determine if the animal exhibited behaviors that could result in a collision with vehicles. Alghamdi et al. [94] implemented YOLOv3 to detect camels on or near roads from images for a vehicle-camel collision system in Saudi Arabia. The model was trained and tested with 600 images and achieved a mAP of 98% at 9 frames per second.

In addition to developing collision warning systems to improve safety, some studies also explored how the warning system would affect drivers’ behavior. Zhao et al. [95] conducted field tests to evaluate the impact of collision types (forward, rear-end, and lateral collision) and warning types (visual warning only and visual plus auditory warnings) in a connected vehicle environment using an in-vehicle omni-direction collision warning system. The findings indicate that driving performance is significantly influenced by collision types, warning types, driver age, and driving experience. Furthermore, it is recommended that such an in-vehicle system should offer visual warnings exclusively for forward collision, whereas it should offer both visual and auditory warnings for lateral and rear-end collisions. Similarly, the effects of warning-based ADAS on driving distraction was investigated using naturalistic driving data from light commercial vehicles [96]. The results demonstrated that active monitoring of warning-based ADAS (1) helped reduce warnings of driver inattention, forward collisions, and lane departures, (2) did not reduce smoking, fatigue driving, and driver yawning, and (3) reduced aggressive driving behaviors tied to harsh acceleration and harsh braking.

Lastly, Table 3 summarizes the application, method, data source, feature, and results of selected studies for crash risk analysis via CV, ML, and DL methods. The major findings and gaps from these studies are as follows:

- When it comes to crash risk analysis using CV, multiple facets are involved, such as crash detection, crash prediction, crash risk analysis, and collision warning systems that take into consideration vehicles, obstacles, and animals.
- There is a trend to apply multimodal data sources into different DL models to perform comprehensive scene analysis and crash risk analysis in real time.
- One significant limitation of current crash risk analysis for ego vehicles is their exclusive focus on images or videos of roadways. To achieve earlier real-time crash prediction, there is a need to integrate information from DMSs (gaze analysis or state monitoring) and road scene analysis into crash risk analysis because many crashes are closely linked to the behavior or state of vehicle drivers.
- The literature review reveals a scarcity of crash risk analysis specifically for trucks or buses.

## 5. Market Scan of AI Tools

Using public information found on company websites or published articles discussing relevant companies, this market scan identified commercially available DMSs that use AI in vehicles. The scan involved finding companies utilizing AI in the context of vehicles, a list of sensors used, and information gathered by the sensors. Over the course of this scan, several roadblocks were encountered due to the proprietary nature of AI algorithms and the novelty of these products in industry. The following sections outline the findings from the market scan and detail the possible gaps due to these roadblocks.

### 5.1. End Users

The three leading use cases of AI technologies in the context of vehicles investigated in this market scan are (1) companies using an AI approach to model and estimate crash severity or crash-related factors, (2) insurance companies using AI technologies to prevent insurance fraud by detecting and analyzing crash events, and (3) companies using AI technologies to analyze drivers’ behaviors and coach or train higher-risk drivers. The leading industry participants in each of the three main use cases included well-known insurance companies such as Progressive and Nationwide, carriers and fleets such as J.B. Hunt and Knight-Swift, and other companies that use information for crash analytics like Tangerine and Field Logix. Initially, the research team contacted the insurance providers, fleets, and crash analytics companies via phone to discuss details unpublished on the website. However, these companies were reluctant to share information regarding third-party relationships or in-house technology via phone or without a written agreement discussing confidentiality. Therefore, the information detailed herein can be exclusively found on public websites or in published articles.

The results from the initial scan indicate that several insurance companies view AI as the future of technology, but only a few are currently implementing it or advertising it on their website. Similarly, many insurance companies do not create their own DMSs or telematics apps, but promote that they can accommodate a large range of providers. For example, Nirvana insurance partners with over 30 providers of telematics or DMSs that the fleets already have installed in their vehicles. Nirvana then takes this information and builds “proactive AI-powered” models to “uncover risk areas” with drivers and hazardous routes and “reduce fraud”. Several other companies follow this model to collect data from a wide range of users. Other insurance companies, such as Progressive and Nationwide, use proprietary algorithms supported by Cambridge Mobile Telematics. However, it was more difficult to find detailed information from these large companies. For example, company websites often direct website users to a contact page to learn more, but usually it is to find out more about purchasing their product rather than divulging more about the functions of their AI algorithms. These factors indicate that industry participants are hesitant to provide much detail about novel technology on their website, but do use key words such as “AI-powered” and “algorithms”, which indicates that they view these technologies as an edge over the competition.

### 5.2. Sensors and Attributes

Although information specifically outlining the capabilities of the AI algorithms was not detailed on most websites, many companies provided specifics about the sensors being used and the attributes the algorithm uses to determine driver state. The most detailed technology provider was Samsara [97]. Samsara stated they use a dual-facing AI dash-cam with an IR light for nighttime driving, an audio recorder, and an accelerometer. The AI algorithm defines distracted driving as events where the driver’s head position is looking away from driving-relevant locations such as the speedometer, mirrors, or forward roadway. Drowsy driving is defined by facial features such as yawning, slapping the face, etc.

After reviewing the leading technology providers for similarities, we can conclude that many companies use dual-facing or driver-facing camera systems to identify objects or facial features in the scene and then use an AI algorithm to determine the state of the driver from these identified objects. When specific information about the sensors was not mentioned, sometimes websites included illustrative photos of the AI in action. The images can include boxes around the driver’s face to show that the CV looks for head position as an indicator of distraction. Another option is highlighting the distracting item, such as a cell phone, to mimic the CV identifying an object in the driver’s hand that is unrelated to driving. An interesting finding is that many leading technology providers use “human review” to validate the decisions made by the AI system. This may be evidence that AI algorithms are not yet to full capability and require human interpretation to further train the algorithms.

The most frequently cited sensor type was camera, followed by accelerometer. The cameras detected attributes such as head position, facial feature detection, hand movement, cell phone use, food/drink, and yawning. The information collected by these sensors was used to predict drowsy and distracted driving. Other driver states were considered, such as aggressive driving, but the former two were most common. Aggressive driving was defined as following too closely, disobeying traffic signals, or making harsh maneuvers. In a heavy-duty vehicle, harsh braking was defined as less than −0.47 g, harsh acceleration was defined as greater than 0.29 g, and harsh cornering was defined as greater than 0.32 g or less than −0.32 g. No websites mentioned intoxication, being under the influence, or any other form of drug use monitoring. The most cited motives for gathering this information were driver coaching, crash prediction or prevention, and seatbelt usage detection by an AI-ML algorithm.

### 5.3. AI Algorithms

The main barrier when conducting the market scan was connecting which AI algorithms were being used by specific industry leaders. It is possible that many of these companies use proprietary algorithms on their driver data to gain a competitive edge in the industry and purposefully leave out details to prevent giving away proprietary information. General descriptions of the AI algorithms were commonly found on informational websites. For example, Lytx’s website [98] defines CV as the system that detects objects and facial features and states that the AI algorithms use this information to determine whether a driver is performing a risky behavior; however, Lytx fails to include details about how these algorithms identify or define driver state, nor do they explain what is considered “safe” driving. The leading DMS suppliers, such as Samsara [97], Lytx [98], Omnitracs [99], and Nauto [100], use verbiage like “advanced edge computing” and “AI-driven processors” but do not explain in detail how the AI algorithms are used, evaluated, or trained. Many companies claim their algorithms “reduce risky driving behaviors” and “decrease crashes”. These claims may be considered methods to evaluate the AI algorithms in an applied context.

Table 4 shows technology companies and the descriptions of their AI algorithm capabilities, including “Company Name”, “AI Capability”, “AI Purpose”, and “AI Purpose Summary”. The “Company Name” is the technology provider being investigated. The “AI Capability” descriptions are taken directly from the company’s website and describe what the AI algorithm is stated to be capable of. For example, Samsara states that AI algorithms use CV for object detection and live scene analysis. The “AI Purpose” descriptions are details from the technology provider’s website that explain what the AI algorithms are being used for. For example, many companies describe using AI algorithms to create a driver profile to determine whether they are a risky driver. The “AI Purpose Summary” column is a synthesized version of the “AI Purpose” description to make it easier to compare the ways companies are using these algorithms. There are five classifications for the AI Purpose Summary:

- Driver Coaching: This classification indicates this company markets their AI algorithm as a way for fleets to identify risky drivers so they can be coached by safety management on proper driving habits. This classification also indicates that the AI algorithm analyzes risky driver behaviors to give drivers a scorecard review.
- Crash Prediction: This classification indicates this company uses an AI algorithm to analyze risky driving behavior and factors in environmental conditions such as weather, time of day, or route to predict whether a driver is at an increased risk for a crash.
- Insurance Claims: This classification indicates a company uses their telematics system with an AI algorithm to exonerate drivers against false claims, reduce insurance costs for drivers classified as “safe” drivers, or mentions reducing insurance costs in some way.
- Crash Reconstruction: This classification indicates this company uses an AI algorithm to reconstruct a crash to determine fault or determine what the driver was doing that may have caused the crash.
- Behavior Prediction: This classification indicates this company uses an AI algorithm to collect driver behavior trends such as seatbelt use during specific times, times they seem drowsy, etc., to determine when a risky driving behavior is most likely.

Although there are many claims made by the industry companies, there are few websites that back up these claims with an evaluation plan or data. In a general sense, a good way to evaluate the effectiveness of the AI might be to corroborate the claims made by industry participants to prevent insurance fraud, reduce crashes, improve driver coaching, and predict risky driving behavior. Similarly, within the field of AI, there are evaluation techniques used to ensure consistency among users. Tian et al. [101] noted the lack of standards when comparing the performance of DMS technologies and their algorithms. Similarly, this paper notes the large inconsistencies among individuals when analyzing facial features such as eyes and mouth when using eye tracking or facial-feature detection algorithms.

**Table 4:** Summary of descriptions used on technology providers’ websites about AI-ML algorithms, their claims, and purposes.

Company Name	AI Capability	AI Purpose	AI Purpose Summary
Samsara [97]	Advanced edge computing, live scene analysis, and object detection.	Coach drivers to improve safe habits. Prevent incidents before they happen by predicting driver risk. Exonerate drivers against false claims.	Driver coaching, crash prediction, insurance claims.
Cambridge Mobile Telematics [102]	AI-driven platform gathers sensor data from millions of devices and fuses them with contextual data to create a unified view of the vehicle and driver behavior	Predict who is at risk of crashes and costly claims (insurance).	Crash predication, insurance claims, crash reconstruction.
Geotab [103]	AI connected sensors capture risky driving events	Predict driver behavior.	Behavior prediction
Orion Fleet Intelligence [104]	AI Capabilities detect driver behavior	Predict driver behavior. Driver coaching.	Behavior prediction, driver coaching, insurance claims.
Lytx [98]	Advanced CV & AI capture and accurately categorize risky driving behaviors	Predict driver behavior. Prevent collisions from happening to protect fleet’s bottom line.	Behavior prediction, crash prediction, insurance claims.
Omnitracs [99]	Intelligent Triggering	Eliminate distracted driving and protect fleet’s bottom line.	Driver coaching.
Trimble [105]	AI technology senses in-cab movementsAI algorithms that can distinguish between driver movements, predict potential scenarios and help reduce collision loss	Advanced algorithms prevent issues and address complex needs like load matching and fuel management. Help reduce accidents, maintain compliance, and potentially avoid costly litigation and insurance costs for fleets.	Crash prevention, driver coaching, insurance claims.
Azuga [106]	DMS captures video and processes them through AI-engine to analyzes each driver-facing video to look for possible distraction events	Protect drivers and business success with the ability to exonerate the fleet when the driver is not at fault. Reduce insurance premiums and accidents. Monitor and alert risky distracted driving behaviors.	Driver coaching, crash prevention, insurance claims.
Zenduit [107]	DMS captures video and processes them through AI-engine to analyzes each driver-facing video to look for possible distraction events	Facial AI Technology can detect crucial driver distraction events with total accuracy. Monitor high-risk behavior to prevent accidents before happening.	Crash prevention, driver coaching.
JJ Keller [108]	AI processor with passive dash cam monitoring	Minimize litigation risk with dash camera technology.	Insurance claims
Blue Arrow [109]	AI & CV uses harsh acceleration, harsh cornering, and harsh braking events to help fleets avoid possible collisions. With AI, unsafe driving behaviors like drowsiness and distracted driving can be monitored and customized to coach drivers	Cameras can improve the safety, productivity, and efficiency of fleets. Manage recordings to protect from insurance fraud and false claims. Correct risky driving behaviors.	Driver coaching, insurance claims.
Fleet Complete [110]	AI on-the-edge processing processes events without the need of a network, allowing event identification to occur quickly and efficiently	Integrate AI-powered video telematics with a fleet management solution to improve the quality of fleet safety programs. Manage poor driving habits and reduce liability costs.	Driver coaching, insurance claims.
Nauto [100]	Predictive AI continuously processes images from the sensor to analyze facial movements and detect unsafe driving behavior	Reduce risk by giving drivers the power to prevent collisions. Makes manager-led coaching more targeted and efficient. Encourages safer driving. Accelerates claims management.	Driver coaching, insurance claims, crash prediction.

Overall, the availability of information regarding AI algorithms used in industry is limited in terms of available details on public websites; without utilizing contacts within the organizations specific information about the way AI is being used is difficult, if not impossible, to find. There are three major gaps found in this market scan:

- Firstly, criteria about how proprietary AI algorithms define driver states such as distracted driving and drowsy driving are still unclear.
- Secondly, there are no evaluation criteria available for each monitoring system, and it is difficult to compare the AI algorithms between companies without understanding how each system defines its variables.
- Lastly, the information gathered about AI algorithms explained the benefits of the technology, such as decreasing crash risk or improving driver coaching, but did not explain how these results were achieved.

These gaps may be due to the intended audience of the websites (i.e., fleet managers, not technology experts) or the proprietary nature of the information being sought. Although these major gaps created barriers in the search, valuable information regarding sensor types, key attributes for defining driver states, trends of industry, and evaluation methods for DMSs used in industry were gathered. For future exploration into this area, an agreement should be reached with relevant technology providers to give specific details with the promise of confidentiality.

## 6. Conclusions

This research conducted a comprehensive assessment of AI tools in driver monitoring and analyzing vehicle-centric SCEs by summarizing technical publications and performing a market scan. The major findings from the assessment are as follows:

- Compared with traditional CV techniques, DL methods improved the performance of image-based driver gaze analysis, driver state monitoring, and SCE analysis in many studies.
- For driver gaze analysis, the image resolution, size and diversity of the training dataset, and the number of gaze zones affected the model’s performance. It is desired to determine which are the critical driver gaze zones to maintain safe driving.
- For driver state monitoring, noncontact-based drowsiness monitoring using CV and DL methods showed better performance than contact-based methods and were cheaper and easier to install.
- The DMSs have a trend to leverage advanced AI models to integrate multi-modal (RGB, depth, or IR) and multi-view (front, top, or side) images of drivers and road scene to compressively analyze SCEs in vehicles.
- One notable limitation in prior studies on the analysis of SCEs is their exclusive focus on images or videos related to traffic or roadways. To achieve earlier real-time crash prediction, it is imperative to incorporate information from DMS (gaze analysis or state monitoring) and road scene analysis into SCE analysis, as identified unsafe driver behaviors and high-risk driver states can serve as early indicators of SCEs, potentially preventing them before they occur.
- Studies involving OEM-integrated DMSs for trucks or buses are absent, as these systems have only recently come online with the advancement of ADAS technologies. As such, the literature review reveals a scarcity of DMS-identified SCEs and of identified crash modification factors from trucks or buses as heavy vehicle-integrated DMS catch up to passenger vehicles.
- The industry is reluctant to share how they implement AI in their DMS in detail, including definitions of different driver states, common evaluation criteria of different DMS, and how AI was used to decrease crash risk or improve driver coaching.

In conclusion, AI methods have demonstrated superior performance in driver monitoring and analyzing SCEs in vehicles compared to traditional CV techniques. Factors like image resolution, training dataset diversity, and gaze zone identification impact model performance. Also, noncontact-based driver drowsiness monitoring, using AI methods, proves more effective and cost-efficient than contact-based approaches. Further, DMSs are adopting advanced AI models, incorporating multi-modal and multi-view images for comprehensive analysis of SCEs. Lastly, the literature review identifies a gap in DMS studies for trucks or buses, and indicates overall industry reluctance to share detailed AI implementation specifics.

It is recommended that future efforts should be implemented in AI applications for driver monitoring and analysis of SCEs for trucks or buses because they are larger, heavier, and behave differently in many aspects during SCEs. Additionally, it is desired to combine DMS information and road scene analysis via AI methods for early real-time crash prediction. Furthermore, to encourage greater transparency within the industry regarding the implementation of AI in driver monitoring and analysis of SCEs, several strategies should be considered in the future, such as establishing industry standards, encouraging research collaboration, and developing and adopting open-source platforms. This initiative offers a valuable asset for academia and industry practitioners aiming to gain a comprehensive understanding of harnessing AI tools using different cameras (color, depth, or IR) to reduce driver errors and enhance driving safety.

## Figures and Tables

**Table 1:** Selected Research of Driver Gaze Analysis.

Paper	Neural Network Type	Camera	Dataset Size	Input Data	Features	Camera Resolution	Training Image Resolution	Accuracy	No of Gaze Zones
Choi et al. [36]	CNN	RGB	35,900	Color image	Detected face image	256 × 256	227 × 227	95%	9
Fridman et al. [31]	Random Forest	Grayscale	1,860,761	Grayscale image	Facial landmarks	800 × 600	N.A.	91.4%	6
Naqvi et al. [37]	CNN	NIR	19,566 and 19,542	Grayscale image	68 face landmarks and ROI of face, left, and right eye	1600 × 1200	224 × 224	92.8% (SCER) and 99.6% (LCER)	17
Vora et al. [38]	SqueezeNet	RGB	47,515	Color image	Upper half of the face image	2704 × 1524	227 × 227	95.18%	7
Wang et al. [34]	Neighbor selection and PLSR	RGB and infrared	50,000	Color and depth image	Head pose and gaze angle	640 × 480	320 × 240	7.5682 in Mean Absolute Error	9
Shan et al. [35]	Random Forest	N.A.	90,791	Color image	Facial landmarks for head and eye features	N.A.	N.A.	94.12%	10
Stappen et al. [40]	InceptionResNetV2	RGB	50,000	Color image	Face + cabin image or facial + Go-CaRD feature	N.A.	150 × 150	71.62%	9
Rangesh et al. [39]	GPCycleGAN and SqueezeNet	Intel RealSense IR camera	336,177	Grayscale image	Landmarks and cropped eye image	640 × 480	256 × 256	80.49%	7
Ledezma et al. [32]	Emgu CV library	RGB and infrared	27,000	Color image	Eye ROI and pupil center coordinate	N.A.	N.A.	81.84%	5
Shah et al. [27]	YOLO-V4 and InceptionResNet-v2	RGB	135,409	Color image	Face image	N.A.	299 × 299	92.71%	10
Kasahara et al. [41]	Self-supervision	RGB-D and Kinect Azure cameras	123,297	Color image	Face image + roadway scene image	N.A.	N.A.	6.2 in Mean Absolute Error	N.A.

Notes: “N.A.” indicates the relative information is not applicable or was not available in the research paper.

**Table 2:** Selected Research of Driver State Monitoring.

Paper	Application	Neural Network Type	Device	Feature	Data	No. of Classes	Results
Vural et al. [51]	Drowsiness detection	Adaboost classifier and MLR	DV camera	Facial actions and head motion	44,640 samples	2	92% accuracy for Adaboost classifier and 94% accuracy for MLR
Reddy et al. [53]		Compressed CNN	Logitech C920 HD Pro Webcam	Image of left eye and mouth	70,000 images	3	89.5% accuracy
Revelo et al. [54]		Landmarks and MLP neural network	Infrared camera	Eye landmarks or eye image	2400 images	2	84% for method 1 and 97% for method 2
Hashemi et al. [55]		CNN	HD webcam camera	Eye image	ZJU and 4185 images	2	96.39% accuracy
Krishna et al. [57]		YOLO-V5 and Vision Transformers	DSLR camera	Face image	UTA-RLDD and 1246 frames	2	95.5% accuracy
Alameen and Alhothali [59]		3DCNN and LSTM	In-car camera and Kinect camera	Frontal and side images	YawDD and 3MDAD	2	>93% accuracy for YawDD and 3MDAD
Lopez et al. [52]	Fatigue classification	AlexNet and SVM	Thermal camera	Face image	5700 images	2	80% accuracy
Zhao et al. [63]	Behavior recognition	Random Forest	CCD camera	Driver side image	SEU	4	88% precision
Yan et al. [64]	Behavior recognition	CNN	CCD camera	Driver side image	SEU	6	97.76% precision
Köpüklü et al. [71]	Driver anomaly detection	MobileNetV2	Depth and infrared camera	Driver front and top images	650 min video	2	0.9673 AUC
Das et al. [12]	Drowsiness and distraction detection	Segmented windows and cascaded late fusion	Physiological sensors, RGB cameras, NIR camera, and thermal camera	Thermal feature vector, facial landmarks, and physiological sensors	Around 420 recordings	2	84% F1-score for drowsiness and 78% F1-score for distraction
Abosaq et al. [65]	Unusual behavior detection	CNN	DSLR camera	Driver video	9120 frames	5	95% precision
Jain et al. [68]	Maneuver anticipation	LSTM	GPS, face camera, and road camera	Videos, vehicle dynamics, GPS, and street maps	Brain4Cars	3	90.5% precision
Hou et al. [67]	Phone usage detection	Mobilenet-SSD	RGB camera	Driving image	6796 images	2	99%
Chang et al. [70]	Drunk driving detection	VGG and Dense-Net	Logitech C310 webcam	Facial image and breath alcohol concentration	20,736 images	2	87.44%

**Table 3:** Selected Research of Crash Risk Analysis.

Paper	Application	Neural Network Type	Data Source	Feature	Results
Chan et al. [73]	Crash prediction	DSA-RNN	Dashcam video	Appearance and motion feature	Predict car crash 2 s earlier with 80% recall and 56.14% precision
Suzuki et al. [75]		AdaLEA	Dashcam video	Global and local feature	Predict car crash 2.36 s earlier with 62.1% mAP and 3.65 s ATTC
Li et al. [79]		Exploratory analysis and association rule mining	Dashcam video and crash report	Temporal distribution of driving scene and fatal crash features	Attention guidance assists CV models to predict fatal crash risk
Choi et al. [76]	Crash detection	CNN and GRU	Dashcam video and audio	Detected cars from image, audio features, and spectrogram image	Car crash detection with AUC = 98.60 for case study 1 and AUC = 89.86 for case study 2
Karim et al. [78]	Crash risk analysis	Multi_Net	Dashcam video	Object detection and segmentation	Generate a likelihood of crash, road function, weather, and time of day to identify crash risk
Shi et al. [77]		CNN and GRU	Kinematic data	Triaxial acceleration	Classify crash, near-crash, and normal driving with 97.5% accuracy
Schoonbeek et al. [82]		RiskNet	Front-facing camera	Intermediate representations of video data	Classify safe and unsafe with 91.8% accuracy
Loo et al. [85]		XGBoosting and RF models	Bus dashcam video	Pedestrian tracking, jaywalking index, and sidewalk railing detection	Pedestrian exposure, jaywalking, crowding, and sidewalk railings are critical to address bus–pedestrian crashes
Sharma and Shah [90]	Collision warning system	HOG and cascade classifier	Camera video	Feature extraction and distance calculation	Achieved 82.5% accuracy for animal detection under speeds of 35 km/h
Rill and Faragó [88]		YOLOv3 and CNN	Spherical camera and smart glasses	Vehicle detection, depth estimation, and TTC calculation	RMSE ≤ 1.24 s for TTC estimation
Venkateswaran et al. [86]		YOLO and Kalman filter	Camera video	Vehicle detection and tracking, distance estimation	Precision ≥ 0.85 for vehicle detection and RMSE ≤ 9.14 for vehicle tracking
Mowen et al. [93]		CNN	Thermal image	Feature maps	Achieved 82% accuracy to classify animal poses
